# The Role of Community Networks in the Transmission and Persistence of *M. tuberculosis* in Urban Africa with Endemic Tuberculosis

**DOI:** 10.1093/ofid/ofaf671

**Published:** 2025-11-04

**Authors:** Ronald Galiwango, Trang Quach, Sarah Zalwango, Samuel Kirimunda, Robert Kakaire, Juliet N Sekandi, Caitlin Williams, Jianing Xu, Frederick Quinn, Liang Liu, Noah Kiwanuka, Christopher C Whalen

**Affiliations:** The African Center of Excellence in Bioinformatics and Data Intensive Sciences, Infectious Disease Institute, Makerere University, Kampala, Uganda; Global Health Institute, College of Public Health, University of Georgia, Athens, Georgia, USA; Public Health Services and Environment, Kampala Capital City Authority, Kampala, Uganda; Department of Microbiology, College of Health Sciences, Makerere University, Kampala, Uganda; Global Health Institute, College of Public Health, University of Georgia, Athens, Georgia, USA; Global Health Institute, College of Public Health, University of Georgia, Athens, Georgia, USA; Department of Infectious Diseases, College of Veterinary Medicine, University of Georgia, Athens, Georgia, USA; United States Army Veterinary Corps, Fort Sam Houston, Texas, USA; Department of Statistics, Franklin College, University of Georgia, Athens, Georgia, USA; Department of Infectious Diseases, College of Veterinary Medicine, University of Georgia, Athens, Georgia, USA; Department of Statistics, Franklin College, University of Georgia, Athens, Georgia, USA; Institute of Bioinformatics, University of Georgia, Athens, Georgia, USA; Department of Epidemiology and Biostatistics, School of Public Health, College of Health Sciences, Makerere University, Kampala, Uganda; Global Health Institute, College of Public Health, University of Georgia, Athens, Georgia, USA

**Keywords:** Africa, contact networks, *M. tuberculosis*, transmission dynamics, tuberculosis

## Abstract

**Background:**

Tuberculosis persists today in many resource-limited countries in the southern hemisphere because unobserved transmission of *M. tuberculosis* occurs in undefined contact networks of infectious cases.

**Methods:**

To study the transmission dynamics of *M. tuberculosis* in an African city with endemic tuberculosis, we built out a sociocentric network in the Lubaga Division of Kampala, Uganda, using the personal networks of 130 index cases and 123 community controls frequency-matched by age, sex, and parish. Clusters of genetically related strains were identified using whole genome sequencing was from 99 isolates of the cases. The social distance between cases with related pairs was estimated from the sociocentic network.

**Findings:**

We found that characteristics of this sociocentric network account, in part, for tuberculosis persistence. These characteristics included highly connected network members, or hubs, where mixing among contacts may occur; predominant transmission among contacts with weak, or distant, ties to the index case; and geographic structural holes in the network that may link cases with these unknown contacts.

**Interpretation:**

These findings suggest that active case finding within the social networks of index cases may result in marginal gains in reducing transmission of tuberculosis. To achieve greater gains, transmission in the community may be reduced through population-based strategies that disrupt transmission in geographic hubs of transmission where mixing may occur between infectious cases and community contacts.

**Funding:**

This research was conducted with support from the National Institute of Health (AI093856, AI147319, P30 AI 68386, D43TW010045, D43TW012481).

## Teaser

The current study used a population-based, novel framework to identify patterns of *M. tuberculosis* transmission through community contact networks. We found that social networks are not sufficient to explain the extent or pattern of tuberculosis transmission in the community. The findings suggest that tuberculosis control interventions that focus on households alone or social networks identified by index cases may produce only marginal gains in reducing the epidemic of tuberculosis.

Tuberculosis persists throughout the world today with its greatest burden in sub-Saharan Africa. This persistence is due to the ongoing transmission of *Mycobacterium tuberculosis* and subsequent progression to tuberculosis disease such that the burden of disease remains high in many urban areas of Africa. This transmission occurs when infectious tuberculosis cases encounter susceptible contacts as they go about their daily lives. These encounters accrue over time to form a contact network around each case that comprises both close contacts within a social network as well as casual and incidental contacts. In regions where tuberculosis is endemic, these contact networks of multiple cases may overlap such that a single sociocentric contact network emerges in the community. This self-organized network may represent the substrate for transmission of *M. tuberculosis.* The structure of this network and the pattern of connections among its members may provide important insights into how tuberculosis spreads through the community and why disease persists.

Transmission of *M. tuberculosis* is intense in households and social networks of index cases [1–3] but accounts for only about 10% to 15% of tuberculous infection in the community [4–6]. Since the advent of molecular epidemiology, tuberculosis transmission has been estimated in cities [7–9], regions [10], and entire countries [11] by identifying clusters of cases with related strains of *M. tuberculosis*. When social network analysis has been used to analyze clustered cases, unrecognized connections between cases, vulnerable populations, and unknown venues of transmission [12–15].

Social networks, however, do not account for the full spectrum of interactions a tuberculosis case may have in the community during the weeks and months before diagnosis [6]. We hypothesize that a broader sociocentric contact network that comprises both known and unknown contacts is needed to delineate transmission of *M. tuberculosis*. By combining molecular epidemiology with the knowledge of how the community is connected, we may better understand the patterns of transmission and persistence in endemic settings.

In this study, we built out a sociocentric network from personal networks of index tuberculosis cases [16] and community members in Kampala, Uganda, an African city with endemic tuberculosis. We then used whole genome sequencing of recovered strains of *M. tuberculosis* to identify clusters of related strains and determine the path length between pairs within the clusters to infer transmission in the network.

## METHODS

The study design and population have been published previously [5, 17] and are summarized. Between June 2013 and January 2017, we conducted a cross-sectional, community-based survey of index tuberculosis cases and their personal networks which included household and known extra-household contacts. We enrolled 130 index tuberculosis cases 15 years and older from community health clinics in the Lubaga Division of Kampala, Uganda. All tuberculosis cases were symptomatic and confirmed with microbiologic tests, including sputum microscopy and mycobacterial culture. For comparison, 123 index controls without tuberculosis were frequency-matched to the index cases based on age, sex, and parish in Lubaga.

Personal networks of index participants were ascertained by interviewers using a two-step approach. In the first step, index participants listed members of their households and individuals living outside the household with whom they had a personal relationship or regular contact over the previous 6 months; these direct contacts are referred to as nearest neighbors (see Glossary of Terms in supplement) in the personal networks. In the second step, these nearest neighbors listed contacts in their own personal networks. We asked informants of each step to describe the types of relations, if any, among their contacts. This sampling strategy delineated the personal networks of index participants to a network path length of two and ascertained the connections among them.

We then constructed a sociocentric network by linking members, or nodes, of the personal networks that appeared in multiple networks. Individuals with common membership were identified and merged using fuzzy string matching of first and last names [18] followed by machine learning and artificial intelligence (Dedupe software, https://dedupe.io/) that relied on name, age, sex, and social role. The quality of matching was assessed by content experts from Uganda who were knowledgeable about naming traditions and social roles.

Infection with *M. tuberculous* was determined using the tuberculin skin test (TST; Tubersol, Sanofi Pasteur Limited, Toronto, Canada) among all study participants except index cases. Infection with *M. tuberculosis* was defined according to standard guidelines [19]. Tuberculous infection was defined as infection with *M. tuberculosis*, either as active tuberculosis disease or as latent tuberculosis infection. Contacts who did not meet definitions for tuberculous infection were classified as uninfected. The diagnostic evaluation for pulmonary tuberculosis and tuberculosis suspects was performed according to current guidelines [20].

For whole genome sequencing, we used methods previously published [21]. Isolates were grown in pure culture and DNA extracted, quantified, and normalized by concentration. Whole genome sequencing libraries were prepared and then sequenced using NextSeq 500 or MiSeq at the Centers for Disease Control and Prevention. The Bactopia pipeline (v3.0.0) was used for quality control, assembly, and annotation of the sequence data using default parameters. The sequences were aligned to the reference strain H37Rv (NCBI CLI v.16.4.4 GCF_000195955) and core single nucleotide polymorphisms (SNPs) called. Isolates were removed with < 75% coverage of the reference genome. Pairwise SNP distances were calculated from the SNP matrix. Of the 130 index cases, 107 were sequenced, and 99 isolates were available for analysis. The demographic and clinical characteristics of index tuberculosis cases did not differ according to whether the isolate was sequenced or not.

### Data Analysis

We used undirected links between network nodes and estimated network statistics including degree, average degree, degree distribution, average path length, diameter, local and global clustering coefficients (See Glossary of Terms). We determined the fit of the degree distribution to the Poisson, exponential, log-normal, and scale-free distributions using goodness-of-fit statistics [22, 23]. We determined the number of fully connected sub-networks (ie, components, see glossary) including the single largest fully connected sub-network, or the giant component. We estimated network communities, or groups within the sociocentric network, by using maps of random walks and density of connections (ie, modularity) [24, 25]. We analyzed network robustness by randomly removing single-degree or high-degree network members and used the size of the remaining giant component as a measure of the likelihood of transmission in the overall network. To assemble and analyze the networks we used the packages of the R statistical software [26].

We used whole genome sequences from 99 isolates to identify genetically similar strain pairs with SNP differences from 0 to 20 SNPs [27, 28]. We chose this range to avoid selection bias in favor of personal network transmission; for presentation, the range was categorized into four ordinal groups based on SNP difference.

To evaluate transmission of *M. tuberculosis* within the sociocentric network, we determined the shortest network path between each pair of index cases with similar strains; case pairs that were not connected were defined as “not reachable”. There were 4 cases involving 7 links for whom we did not have relational information; we classified these links as not reachable.

For each case pair, we classified the links between cases as strong or weak. We evaluated link strength in three complementary ways. First, we considered a path length of 1 or 2 from the index participant as a strong link; we used this criterion because it reflected the way we assembled the personal networks of cases and their controls. Second, link strength between pairs was estimated as the proportion of observed links among all nearest neighbors of the nodes of each pair [29]; this measure of link strength was calculated for all possible pairs in the network. Third, we determined how often case pairs were located within the same or different network communities. Since network communities represent groups with higher density of links among members of the group than to members of the network, we considered membership in a community as a type of link strength.

We used a Bayesian transmission model to reconstruct the transmission network by integrating genomic data with temporal information about disease occurrence [30]. Unlike methods that infer a transmission tree from a phylogenetic tree [31], the Bayesian model estimates transmission networks directly from genomic data. The model explicitly incorporates the latent period and distinguishes between symptom onset and time of infection, thereby improving the accuracy of inferred transmission dynamics and epidemiological parameters. It assumes a homogeneous effective population size across hosts, which keeps the within-host coalescent process stable even when intermediate hosts are missing, allowing the model to handle incomplete sampling. Since the Bayesian transmission model accounts for missing ancestors, the connections in the estimated transmission network could represent either direct or indirect transmissions. To distinguish these, we constructed the Bayesian 95% confidence interval [0, *c*] for the number of SNPs associated with each of the 98 edges in the estimated network. Direct transmissions were identified if the observed number of SNPs is less than or equal to the upper bound *c*.

## RESULTS

We enrolled 130 tuberculosis cases and 123 age- and sex-matched community controls without tuberculosis. Compared with controls, index tuberculosis cases had a higher proportion of HIV-1 infection, were less likely to be married, and had a greater number of contacts with latent tuberculosis infection (Supplementary Table 1).

**Figure 1.**
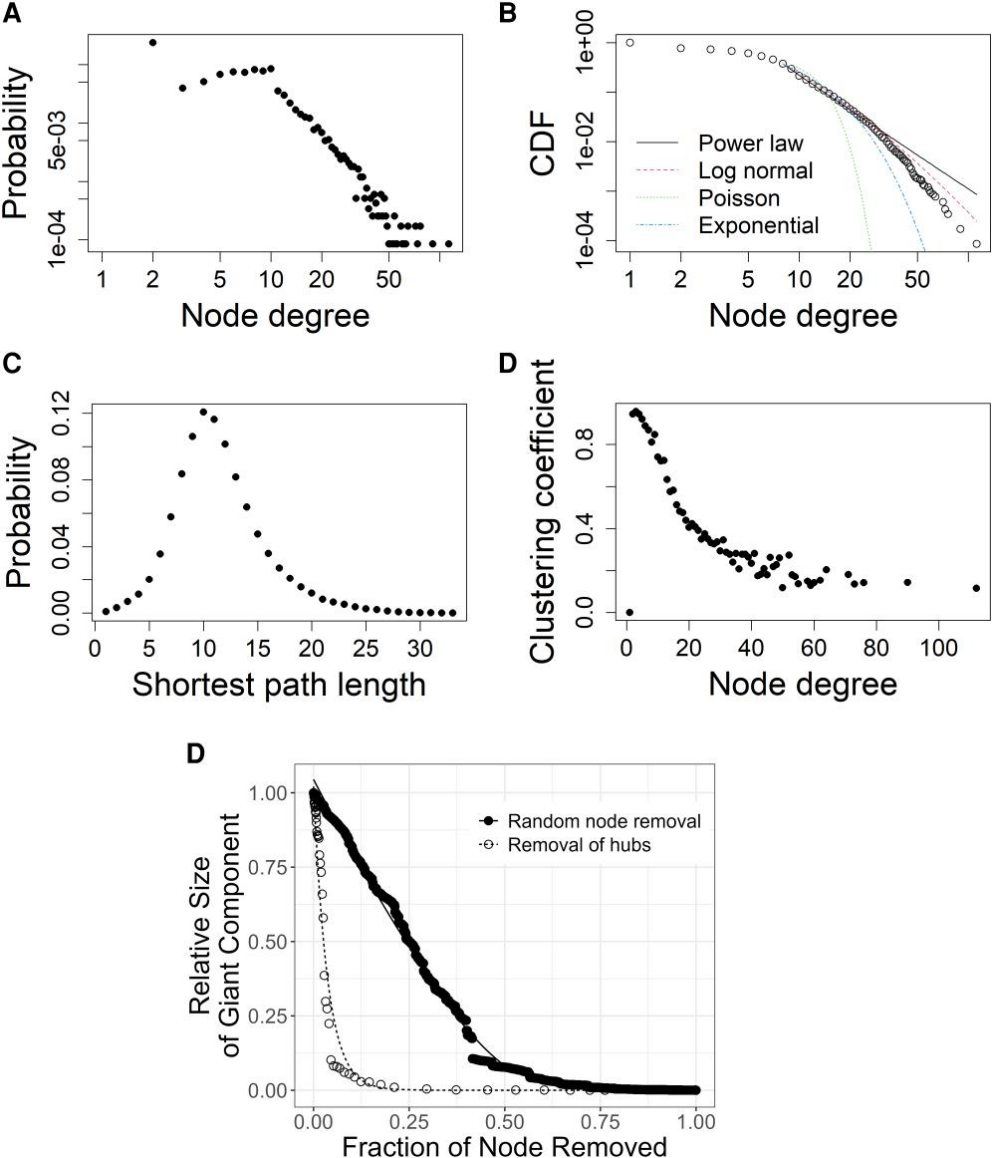
(*A*), Distribution of connections (ie, degree distribution), (*B*), Cumulative distribution function (CDF), (*C*), Shortest path length distribution, (*D*), Local clustering coefficient, and (*E*), robustness assessment for sociocentric network of 11 739 members containing 130 tuberculosis cases and 123 matched controls. (Figures *A* and *B* presented on log-log scale). Exponential, Poisson, and scale-free distributions did not fit the observed distribution (*P* < .001) using goodness-of-fit test developed by Clauset and colleagues [32]. Regression lines were fit using loess or non-linear regression. Figure *A* is the degree distribution, or the number of connections per person, on a logarithmic scale; Figure *B* illustrates that this degree distribution, or pattern of connection, is most consistent with a log-normal distribution, which is produced by the interaction of numerous independent events; Figure *C* indicates that the average path length between any two nodes in the network is about 10 steps; Figure *D* indicates that the clustering of nodes reduces as the number degree increases; Figure *E* show the relative size of the giant component (*y*-axis) following node removal compared with the complete network including all nodes. The relative size of the giant component is a measure for the probability of transmission within the network.

The sociocentric network comprised 11 739 unique nodes and 40 874 links (Supplementary Figure 1). The mean number of links for each node was 7.0, and the distribution of these links was most consistent with a log-normal distribution (Figure 1*A* and *B*). The network was sparsely connected (link density = 0.0006), wide (diameter = 33 and average path length = 11.5; Figure 1*C*), and clustered (global clustering coefficient = 0.51), though the clustering decreased as the degree increased (Figure 1*D*). In the overall network, the nearest neighbors of a given pair of nodes were rarely linked (1.4%; 95% CI: 1.2, 1.6), indicating a paucity of this type of strong link within the network.

The network was decomposed into 47 components. The giant component comprised 9885 (84%) nodes (Supplementary Table 2), including 92 of the index cases and 101 of the index controls; the remaining 38 index tuberculosis cases were distributed in one component of 177 nodes with 3 cases, one component of 138 nodes with 2 cases, one component of 12 nodes with 2 cases, and 31 components with 1 tuberculosis case each. No cases were found in 12 components.

Although the network did not follow a strict power law [33], the second moment of the degree distribution was much larger than the average degree (93.4 vs 6.9), indicating the distribution was not exponentially bounded. The network was robust to the random removal of single nodes (Figure 1*E*), meaning that transmission could be sustained through remaining links; 91.9% of the single nodes had to be removed before the transmission links in the giant component were extinguished. In contrast, when network nodes with numerous connections (ie, hubs) were removed sequentially, the giant component and the potential for transmission, diminished rapidly.

### Networks of Index Tuberculosis Cases and Community Controls

Within the sociocentric network, the sub-networks of index tuberculosis cases were connected in ways that would promote transmission of *M. tuberculosis*. Case networks had fewer members (55 vs 64 nodes), were more compact (diameter 3.7 vs 3.8), were more connected (higher edge density of links: 0.13 vs 0.10), and were more clustered (average local clustering coefficient: 0.93 vs 0.86; Table 1). Despite the more compact and connected networks of cases, a greater proportion of case pairs did not have a definable path between them when compared with control pairs (41.4% vs 30.8%) and were less central in their networks (betweenness: 0.29 vs 0.42). Although the link strength was weak between most pairs of network members, a greater proportion of index cases had strong links to their nearest neighbors than did controls (1.9% vs 0.9%).

**Table 1.** Comparison of Personal Network Characteristics of Tuberculosis Cases and Their Matched Controls

	Personal Networks of TB Cases (*N* = 124)^a^	Personal Networks of Controls (*N* = 123)	*P* value^b^
No. of nodes, median (IQR)	55 (31, 98)	64 (45, 82)	.825
No. of edges, median (IQR)	185 (96, 339)	184 (98, 323)	<.001
Edge density, median (IQR)	0.128 (0.071, 0.252)	0.096 (0.059, 0.134)	<.001
Average path length, median (IQR)	2.50 (2.01, 2.79)	2.64 (2.42, 2.96)	<.001
Diameter			
2	5 (4.0)	1 (0.8)	.069
3	33 (26.6)	23 (18.7)	…
4	86 (69.4)	99 (80.5)	…
Global clustering coefficient, median (IQR)	0.56 (0.49, 0.64)	0.55 (0.46, 0.61)	.02
Local clustering coefficient for all nodes in individual network, median (IQR)	0.93 (0.48, 1.00)	0.86 (0.02, 1.00)	<.001
Degree for index, median (IQR)	17 (10, 28)	16 (10, 29)	.498
Degree for all nodes in personal network, median (IQR)	7 (4, 9)	6 (2, 9)	<.001
Closeness for index, median (IQR)	0.61 (0.57, 0.67)	0.59 (0.55, 0.64)	.034
Betweenness for index, median (IQR)	0.29 (0.12, 0.52)	0.42 (0.21, 0.63)	< .001

See Glossary of terms for definition of network characteristics.

^a^There are 6 TB cases without social network information and were excluded from this analysis. The Mann–Whitney U test is used to compare the distributions of continuous variables; the Chi-square test is used to compare categorical variables.

^b^ *P*-value adjusted for age, sex, income, and HIV status using linear (continuous variables) or Poisson (count data, categories of diameter).

### Transmission of Tuberculosis Within the Sociocentric Network

**Figure 2.**
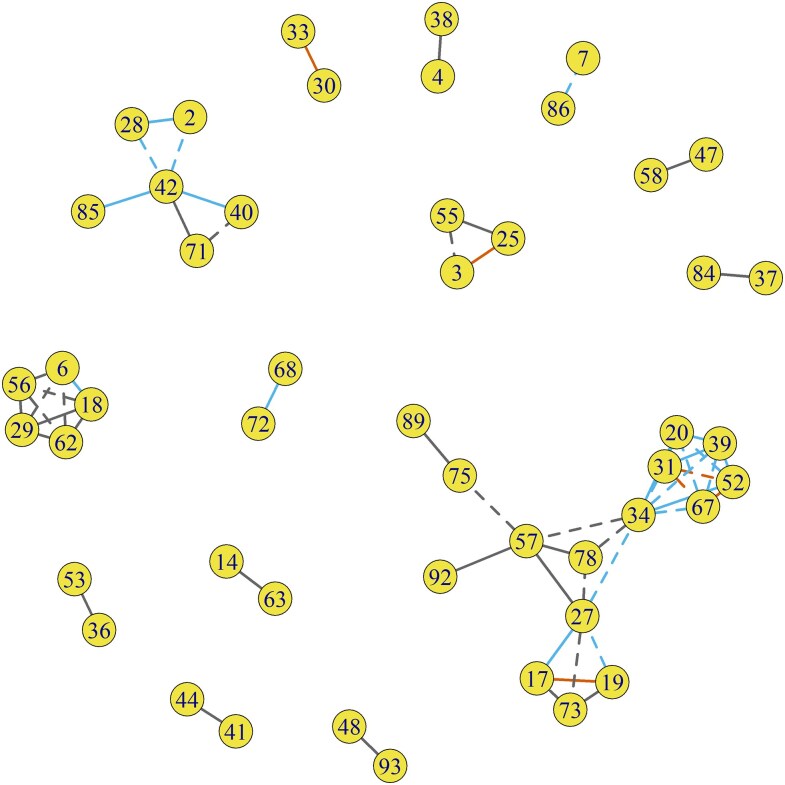
Fourteen clusters of tuberculosis cases with SNP difference ≤ 20 where edges indicate direct or indirect transmission and categorized path length within the sociometric network. Solid edge indicates direct transmission; dashed edge indicates indirect transmission. Red indicates a path length of 2 or less, sky blue indicates path length of 3–14, gray indicates an unobserved.

**Figure 3.**
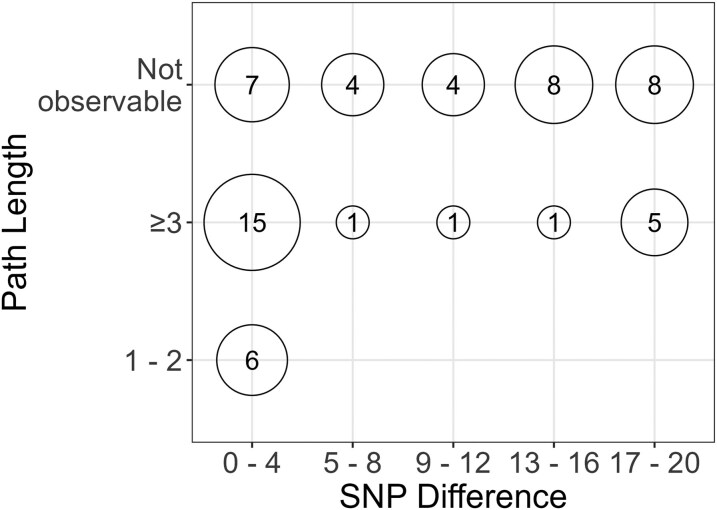
Number of cases pairs according to category of SNP difference and network path length (number of case pairs shown within circles).

In the sociocentric network, 49 cases formed 14 clusters when selecting case pairs using a SNP difference of 20 or less (Figure 2). The cluster sizes ranged from 2 to 15 and contained 60 pairs of cases that were spread among 35 of the 47 network components. Of the 28 pairs with SNP difference < 5, 6 pairs (21.4%) had strong links with a path length of only 1 or 2, whereas the remaining 22 pairs (78.6%) were connected by weak links with paths lengths of 4 or greater, or no observed links (Figure 3). All case pairs with SNP differences of 5 or greater were connected via weak links.

The patterns of clustering show how some cases may have influenced, or bridged, transmission across several different groups in the network to facilitate transmission. For example, in the cluster of 15 cases (Figure 2), 3 cases (case numbers: 27, 34, 57) had high betweenness (0.38, 0.49, and 0.36, respectively) and may have spread related strains between two groups belonging to disparate network communities. Similarly, one case (case number 42) in the cluster of 6 cases had a high betweenness (0.8) and connected at least three unconnected sub-networks.

**Figure 4.**
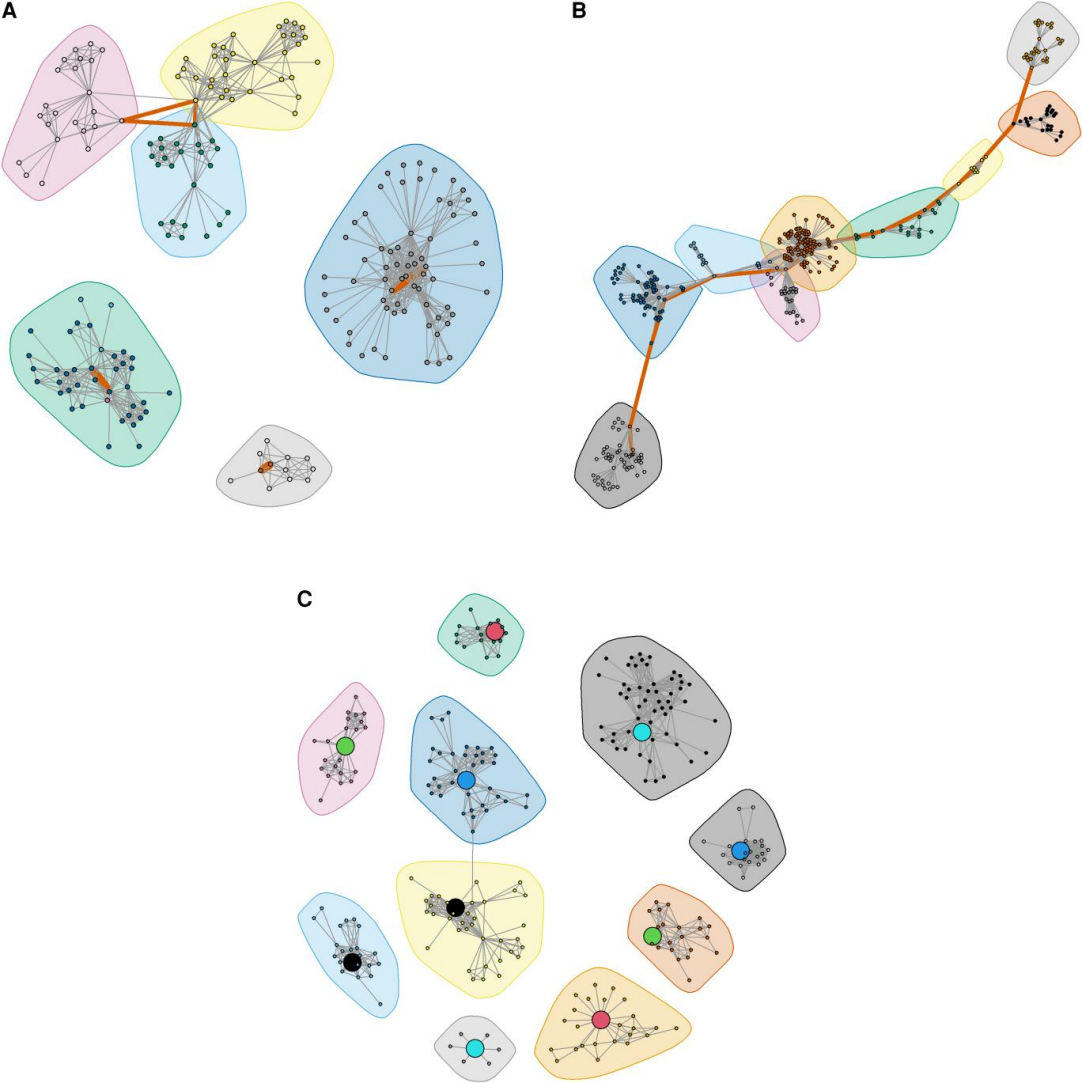
Distribution of case pairs across network communities according to path length. Paths shown in red. (*A*), The 6 pairs of tuberculosis cases with path length of 1 or 2. Three pairs of cases occupy three distinct communities; three pairs within a triad of cases connect across three network communities. (*B*) An example of one pair of tuberculosis cases found in the giant component with a path length of 12 that crosses 9 overlapping communities. (*C*) 5 of 24 pairs with no observable path between them; in each pair (indicated by same circle color), the cases are found in separate and often distinct network communities. The lack of connection indicates a structural hole in the network.

Among the 60 pairs, the Bayesian transmission model estimated 31 direct and 29 indirect transmissions events (Supplementary Figure 2). Of all pairs, only 6 pairs had a path length of 1 or 2 (10%, 95% confidence Interval: 4.7%, 20.2%; Figures 2 and 3). Three pairs of cases occupied three distinct communities; three pairs within a triad of cases connect across three network communities (Figure 4*A*). In 23 pairs (38.3%, 95% CI: 26.7%, 51.1%), the path lengths ranged from 4 to 14, indicating a series of links between nearest neighbors in the network, but no direct link between cases. The shortest path length for each of these pairs passed through 3 to 11 network communities, indicating the opportunity for interaction with contacts from various communities (Figure 4*B*). In 31 pairs (51.7%, 95% CI: 39.4%, 63.8%), no path could be found between the two cases, indicating that the members of the pair were in different, unconnected components of the network (Figure 4*C*). The distribution of path length did not differ between pairs with direct transmission and those with indirect transmission (*P* = .57, Fisher's exact test). The proportion of strong links between cases was similar across scales of the network; the proportion of pairs with a path length of 1 or 2 was 10% in the entire transmission network, 15% in the giant component, and 13% in the largest cluster of 15 cases. Of the 29 indirect transmissions, 17 (58.6%) involved a chain of transmission with at least one intermediary strain between a case pair.

## DISCUSSION

We found that three features of a community network may explain, in part, the persistence of tuberculosis in large African cities with endemic tuberculosis [34]. These features were a degree distribution with some highly connected members, the predominance of weak links between members of the sociocentric network, and evidence for structural holes where co-location may occur among members with weak links. The degree distribution found in this study was most consistent with a heavy-tailed, log-normal distribution; this means that the distribution had a higher variance in degree because it included a preponderance of highly connected network members. The higher variance may favor transmission from infectious cases, even when the probability of transmission is low [35, 36]. Furthermore, the presence of high degree members ensures the robustness of the transmission network to interventions that remove individual members, such as active case finding for tuberculosis [23]. This pattern of network topology is found in many cities around the world [37], so in a setting of endemic tuberculosis, the frequency of high degree nodes may allow for co-location and mixing that drives transmission of *M. tuberculosis*.

In human networks, information flows more efficiently through weak links than strong links [29]. If we view the genome of *M. tuberculosis* as a unit of information, then we have a model that informs testable hypotheses about transmission through weak links in the contact network of tuberculosis. We found that the sociometric network comprised mostly weak links, where the average shortest path between randomly selected individuals was 11 and the nearest neighbors of those individuals were themselves rarely linked. Although transmission did indeed occur through smaller personal, or social, networks with strong links, this pattern accounted for only about 10% of the observed transmission in the entire network; the remaining 90% occurred through weak links with casual or incidental contacts within the contact network. These findings are consistent with our previous studies of tuberculous infection that show only 11% of infection in this community can be attributed to transmission within social networks [6].

The transmission among weak links often extended across multiple network communities through members who acted as bridges between communities [38]. This bridging was seen between case clusters where well-connected influential cases with high network betweenness were connected to other network communities. The effect of weak links also scaled across the network from the single largest cluster of 15 cases to the giant component, and finally to the entire sociocentric network.

Like other molecular epidemiology studies of tuberculosis, we used pairs of index cases that were linked through an undetermined microbial network [27, 39] to infer transmission. Unlike these studies, we measured a sociocentric network and evaluated the path lengths between observed cases with related strains. We found that network paths could not be identified in 52% of the case pairs, despite detailed and systematic network ascertainment. These differences between the observed links in the microbial network and the observed links in the contact network suggest that there were structural holes in the sociocentric network, that is, unmeasured sub-networks that would connect members of the network though venues or neighborhoods. Since the transmission of *M. tuberculosis* occurs between infectious cases and their susceptible contacts that co-locate and share airspace, the transmission events must be temporally and geographically discrete. This type of co-location may occur in local neighborhoods [40–42] or in social settings such as schools [43, 44], bars [6, 41, 45], places of worship [46], prisons [47], hospitals and clinics [48, 49], and other congregate settings [50]. For this reason, we propose that geographic locations visited by tuberculosis cases may represent these structural holes and may account for the unlinked pairs of cases observed in the network. In these locations, cases may interact with casual or incidental contacts that are part of different network communities [51, 52].

This study has potential limitations. One limitation is the incomplete ascertainment of the contact networks of each index case. As a result, the full sociocentric network may not include all possible paths between individuals. This potential selection bias would affect our conclusions if the missing paths overrepresented strong links between index cases and their contacts. We believe that this limitation is minimal because we ascertained strong links directly from informants through structured interviews with temporal, relational, and geographic prompts [53]. Unnamed contacts were likely casual or incidental contacts and unknown to the informant, and hence were contacts with weak links. One caveat to this interpretation is if the informant withheld confidential contacts for privacy. Given that our research team had earned the trust of the community over years of engagement and our systematic approach to network ascertainment, we believe that this issue is uncommon and would not alter the conclusions.

Another potential limitation of the study may be sampling bias. Since complete networks cannot be readily known, samples must be taken to understand the structure of the network. The type of sampling, however, may lead to biased estimates of network properties [54]. Preferential sampling, such as respondent-dependent sampling, may over-represent high degree nodes, enhance community structure [55], and lessen estimates of the degree exponent, betweenness, and assortativity [56]. Although our study may be subject to some of the biases identified in simulations, our sampling design was not evaluated in these studies and may, in fact, address some of the concerns about bias.

Another type of selection bias may have occurred. Since we enrolled symptomatic cases with microbiologically confirmed pulmonary disease, we may have sampled cases that were more infectious than asymptomatic or subclinical cases, leading to an over-estimation of the proportion of transmission through weak links. The effect of this potential bias may be minimal, however, because subclinical disease may be as infectious as symptomatic disease [57, 58].

The findings of this study put into context the effectiveness of current tuberculosis contact investigations in an endemic setting. Since contact tracing relies on the self-reported, known contacts of index cases, it preferentially identifies the contacts with strong network links within the social network. Contact investigation within these social networks may systematically miss the casual or incidental contacts that are weak links in the broader contact network. Moreover, the robust structure of the network may promote transmission of *M. tuberculosis* in the undefined contact network where weak network links predominate. To reduce transmission in the community in African cities with endemic tuberculosis, we suggest that population-based strategies tailor interventions that target geographic hubs of transmission, where contact networks may be most vulnerable. Extending the relational social network to bipartite networks that include locations of contact and mobility may help to identify these geographic transmission hubs.

## Supplementary Material

ofaf671_Supplementary_Data

## Supplementary Data

Supplementary materials are available at Open Forum Infectious Diseases online. Consisting of data provided by the authors to benefit the reader, the posted materials are not copyedited and are the sole responsibility of the authors, so questions or comments should be addressed to the corresponding author.
