# Supplementary material for: The Role of Community Networks in the Transmission and Persistence of M. tuberculosis in Urban Africa with Endemic Tuberculosis
Source: Open Forum Infect Dis. 2025 Nov 4;12(11):ofaf671. doi: 10.1093/ofid/ofaf671 (PMC12628501; doi:10.1093/ofid/ofaf671)
Supplement: ofaf671_Supplementary_Data [file ofaf671_supplementary_data.docx]

**Supplemental Table and Figures**

| Supplemental Table 1: Demographic and social characteristics of index cases and their matched controls. | | | |
| --- | --- | --- | --- |
| Characteristic | cases (N=124*) (%) | Controls (N=123) (%) | P-value* |
| Sex  Male  Female | 85 (68.5)  39 (31.5) | 84 (68.3)  39 (31.7) | 1.000 |
| Median age (range) | 28 (23, 36) | 30 (24, 38) | 0.159 |
| Marital status  Married  Not married | 58 (46.8)  66 (53.2) | 90 (73.2)  33 (26.8) | <0.001 |
| Education  ≥ High school  < High school | 75 (60.5)  49 (39.5) | 73 (59.3)  50 (40.7) | 0.202 |
| Income (USD)  ≥ $56  < $56 | 35 (28.2)  89 (71.8) | 49 (39·8)  74 (60·2) | 0.073 |
| HIV status  Positive  Negative  Missing | 20 (16.1)  99 (79.8)  5 (4.0) | 6 (4.9)  117 (95.1)  0 (0.0) | 0.001 |
| Median number of contacts with tuberculous infection (IQR) | 5 (3, 6) | 4 (2, 5) | <0.001 |
| *The P-value is for comparison between characteristics of index cases and index controls. The Mann–Whitney U test is used to compare the distribution (the medians). The Chi-square test is used to compare categorical variables. Data was missing for 6 index cases who were retained because they provided strains for whole genome sequencing. | | | |

| Supplemental Table 2. Distribution of tuberculosis cases, clusters, and case pairs with related strains (SNP difference ≤ 20) across network components | | | | | |
| --- | --- | --- | --- | --- | --- |
| Components with TB cases | Size  (nodes)  (N) | TB  Cases  (N) | Available  sequences  (N) | Clusters  (N) | Pairs  (N) |
| Network | 11,739 | 130 | 99 | 14 | 60 |
| Component 1 – giant component | 9885 | 92 | 65 | 5 | 27 |
| Component 2 | 177 | 3 | 2 | 1 | 1 |
| Component 3 | 138 | 2 | 2 | 0 | 0 |
| Component 4 | 12 | 2 | 2 | 1 | 1 |
| Components 5 – 47  Multiple, different components | 7 - 117 | 31 | 28 | 11 | 31 |

**`**

| Supplement Figure 1. Full sociocentric network with 11,739 nodes comprising contacts of 130 index tuberculosis cases and 123 neighborhood controls (Personal networks of index cases shown in cerise, red; personal networks of controls shown in jade green.) |
| --- |
| 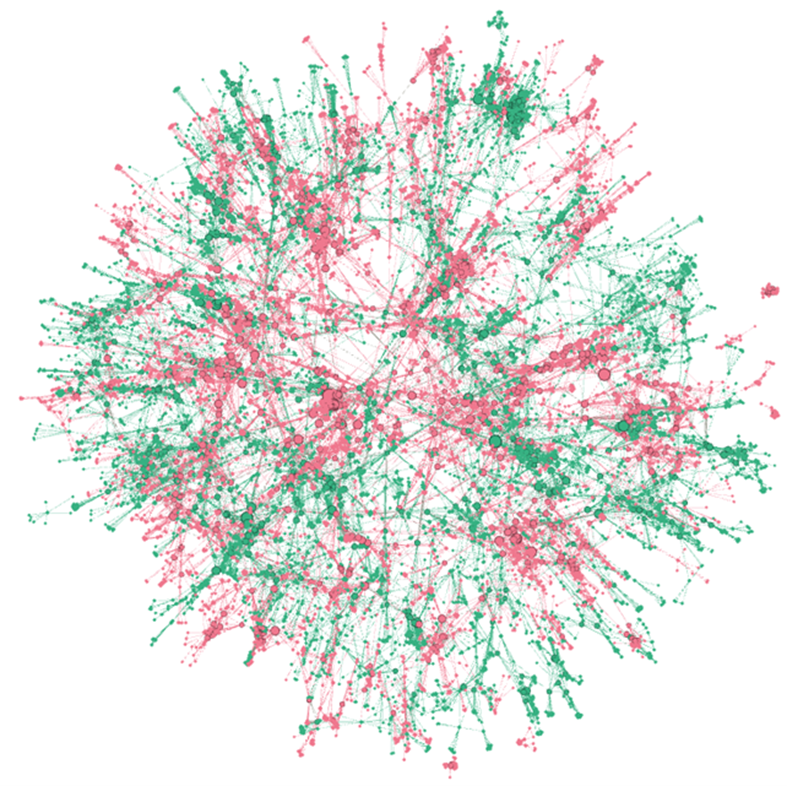 |

| Supplement Figure 2. The Bayesian estimate of the transmission network for 99 tuberculosis cases. The estimated network consists of 98 edges with each edge representing a transmission event. The direction of each transmission is indicated by an arrow. In this network, 31 direct transmissions are highlighted in red edges. |
| --- |
| 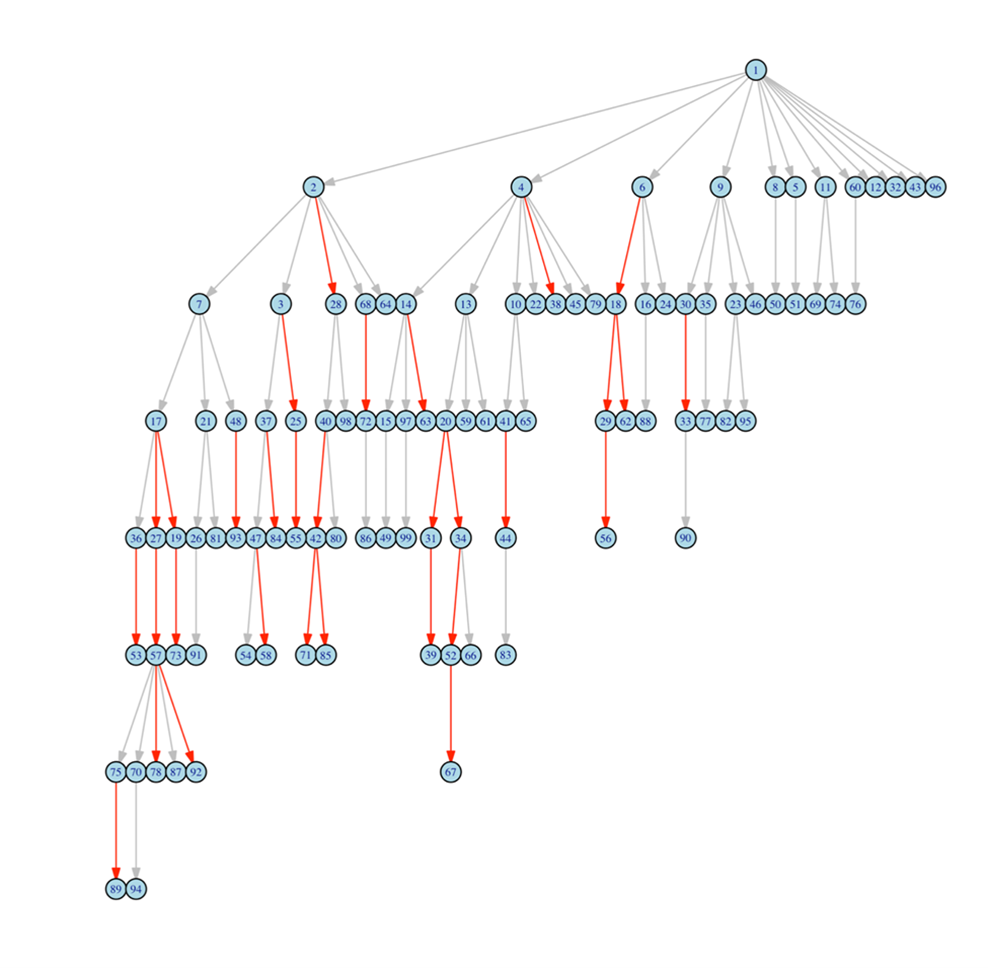 |

Glossary of terms in network analysis

| Network Terminology | Definition |
| --- | --- |
| Node | Member or actor in a network |
| Link | Connection between nodes, also called edge or tie |
| Network size | Number of nodes in a network |
| Degree | Number of links a given node has |
| Average degree | The average number of links each node has in the network |
| Degree distribution | Frequency histogram of degree for a network |
| Density | Proportion of observed links relative to all possible links |
| Path | Sequence of links between nodes in a network |
| Path length | Number of links in a path |
| Average shortest path length | Average of the shortest paths between all pairs of nodes in a network |
| Network diameter | Longest of the shortest paths between all nodes in network |
| Nearest neighbors | Nodes with a path length of one to a given node |
| Local clustering coefficient | Links between the nearest neighbors of two unique nodes |
| Global clustering coefficient | Overall connectivity of nodes in a network |
| Component | Sub-network in which all nodes are connected either directly or indirectly |
| Giant component | Largest component in the network |
| Network community | Sub-network wherein nodes are more connected to other nodes in the sub-network than to nodes outside of the sub-network |

1. Kolaczyk, ED, Csardi, GSpringer Nature. Statistical analysis of network data with R. Second. Cham, Switzerland: Springer Nature; 2020.
2. Luke D. A User’s Guide to Network Analysis in R. NewYork: Springer; 2015.
